# Adverse childhood experiences are associated with a higher risk for increased depressive symptoms during Covid-19 pandemic – a cross-sectional study in Germany

**DOI:** 10.1186/s12888-022-04177-7

**Published:** 2022-08-11

**Authors:** Vera Clemens, Franziska Köhler-Dauner, Ferdinand Keller, Ute Ziegenhain, Jörg M. Fegert

**Affiliations:** Hospital of Child and Adolescent Psychiatry/Psychotherapy, University of Ulm, Steinhövelstraße 5, 89075 Ulm, Germany

**Keywords:** CoVid-19, Pandemic, Adverse childhood experiences (ACEs), Depression, Public mental health

## Abstract

**Background:**

Covid-19 pandemic has been profoundly affecting people around the world. While contact restrictions, school closures and economic shutdown were effective to reduce infection rates, these measures go along with high stress for many individuals. Persons who have experienced adverse childhood experiences (ACEs) have an increased risk for mental health problems already under normal conditions. As ACEs can be associated with a higher vulnerability to stress we aimed to assess the role of ACEs on depressive symptoms during the Covid-19 pandemic.

**Methods:**

In a cross-sectional online survey, 1399 participants above the age of 18 years were included during the first lockdown in Germany. Via two-way repeated measures ANOVA, differences in depressive symptoms before (retrospectively assessed) and during the pandemic were analyzed. Linear regression analyses were performed in order to identify predictors for increase of depressive symptoms.

**Results:**

Compared to prior to the Covid-19 pandemic, depressive symptoms increased among all participants. Participants with ACEs and income loss reported about a stronger increase of depressive symptoms. Other predictors for increased depressive symptoms were young age and a lack of social support.

**Conclusions:**

Based on these results, ACEs are a significant predictor for an increase in depressive symptoms during the pandemic, indicating that personss with ACEs may be a risk group for mental health problems during the current and potential later pandemics. These findings underline the relevance of support for persons who have experienced ACEs and may help to provide more targeted support in possible scenarios due to the current or possible other pandemics. Besides, economic stability seems to be of prior importance for mental health.

**Supplementary Information:**

The online version contains supplementary material available at 10.1186/s12888-022-04177-7.

## Introduction

The novel severe acute respiratory syndrome coronavirus-2 (SARS CoV-2) pandemic has been profoundly affecting people around the world. Contact restrictions, school closures and economic shutdown have proven to be effective reactions to fight against increasing numbers of Coronavirus disease 19 (Covid-19) cases and fatalities [1, 2]. However, this success comes with a dark side. Negative consequences of measures such as “social distancing” and quarantine for mental health have been discussed in reviews [3–6], and confirmed by studies [7–10].

While in most countries, lockdown phases have ended, currently, numerous areas face a still high numbers of Covid-19 infections. Moreover, the occurrence of new pandemics is expected in the next decades. Because depression is even under normal conditions the third leading cause of disability (as measured by years lived with disability; YLDs) [11], an increase can be expected triggered by the challenges of the pandemic. Consequently, to identify predictors of an increase of depression is of major public health interest.

Existing literature suggests that loneliness [12] and less social support [13, 14] are associated with depressive symptoms during the pandemic. Economic stressors were shown to predict depressive symptoms during the pandemic [15]. Younger age is associated with increased mental health problems during the pandemic [9, 16]. Preexisting psychiatric disorders were also identified to predict depressive symptoms [17, 18].

Another predictor for poorer mental health during and in the aftermath of the pandemic may be adverse childhood experiences (ACEs) [19]. Even in more normal times, ACEs are associated with psychosocial and economic impairments, a significant reduction in quality of life, risky behavior and increased morbidity due to both, mental and somatic health problems [20–26]. Importantly, evidence for moderate to strong associations between ACEs and depression, just as for other major psychiatric disorders, were numerously shown in prospective and retrospective studies [20, 25–27]. Lower social support, associated to the experience of childhood adversity [28], was shown to mediate the association between ACEs and mental health [29, 30]. ACEs are associated with lower household income, but also with financial wellbeing and financial literacy [31].

The experience of adversity during childhood is associated with heightened neural response to signals of threat [32], increased emotional reactivity and decreased emotion regulation [33]. Based on these facts, it seems not surprising that ACEs were found to be associated with higher depressive symptoms [10, 34] and emotional exhaustion [35] during the pandemic. However, as ACEs are known to predict depressive symptoms, in order to assess the role of ACEs during the pandemic, change of depressive symptoms compared to before the pandemic has to be assessed. Previously, we have found ACEs to be associated with a stronger decrease in quality of life, general health status, dysfunctional coping strategies [35, 36] and a higher risk for intrafamiliar problems [35, 37, 38]. A higher stress-vulnerability and a decrease of emotion regulation [32, 33] was shown for people who have experienced ACEs, affecting coping of stressful situations.Here, we hypothesized that childhood adversities predict an increase of depressive symptoms during the pandemic. Therefore, in an online survey, we have assessed depressive symptoms before the pandemic retrospectively and current depressive symptoms during the pandemic. Moreover, we aimed to identify other predictors increasing depressive symptoms in order to help identifying high risk groups and developing targeted care.

## Methods

### Study design

Using the platform Unipark, we have conducted a cross-sectional online survey which was available from May 18th–July 21th 2020. The first lockdown in Germany began on March 23, 2020 and ended with gradual relaxations. First schools reopened on April 22, openings of schools and kindergartens stretched to the end of June 2020. We distributed information on the survey by our homepage, social media and print media and existing mailing lists from other studies and interested parties.

### Ethics

Electronic informed consent was obtained from each participant prior to starting the survey. Information on the study and data analysis were given. Participation was voluntary and anonymous. Participants could withdraw from the survey at any moment without providing any justification. The study was conducted in accordance with the Declaration of Helsinki. After consultation with the ethics committee of the University of Ulm, the committee officially stated that there is no requirement for an ethics vote due to the anonymous character of the study.

### Measures

Socio-demographic questions covered among others age, gender, educational level, occupation, marital status, number of persons under 18 years in the household and number of own children. Covid-19 associated questions included and whether the household income decreased by more than a quarter since the beginning of the CoVid-19 crisis. Moreover, it was included whether the participant has been working in a system-relevant work. In Germany, this term refers to people working in jobs that were not paused during the first lockdown in spring 2020 and not affected by working from home, such as personnel in medical institutions, supermarkets, police, etc.

The adverse childhood experiences were assessed using the German version of the Adverse Childhood Experiences Questionnaire, a standard tool for retrospective assessment of ACEs with satisfactory internal consistency (Cronbachs α = 0.76) [39].

Depressive symptoms were assessed by the eight-item Patient Health Questionnaire depression scale (PHQ-8), an established and valid diagnostic and severity measure for depressive disorders [40]. Additionally, we included one item on alcohol and tranquilizer use asking the question: “When everything becomes too much for me, I resort to alcohol or tranquilizers”. Social support was assessed by the question “I have people with whom I can talk about my problems and who understand me”, answers on a scale between [1] stands for "does not apply at all", [10] stands for "applies completely. The assessment of depressive symptoms before the pandemic were assessed retrospectively, as our study had a cross-sectional design.

### Data analysis

All statistical analyses were performed with SPSS version 21. The prevalence rates were determined using descriptive analyses, only valid cases were included. Valid numbers are given for each analysis.

A two-way repeated measures ANOVA was used to test differences in depressive symptoms before (retrospectively assessed) and during the pandemic (main effect time), between groups with different numbers of ACEs (main effect ACE) as well as income loss due to the pandemic (main effect income loss) and a differential effect of ACEs on the increase of depressive symptoms over time (interaction effect time x ACE). The number of ACEs were categorized for this analyses into three groups: 0 vs 1–3 vs 4 and more ACEs.

Linear regression analyses were performed in order to identify predictors for increase of depressive symptoms. As dependent variable, the difference from the PHQ8 total score before and during lockdown was used. Thus, if the PHQ-8 score was higher during lockdown, the difference is negative. If the score was higher compared to before lockdown, the difference is positive. In a first block, gender and age at the point of entry into the institution were analyzed as predictors. In a second block, number of ACEs were included. In a third block, the additional variables systematically important work, decrease of household income since the beginning of the pandemic, living alone, social support, and pre-existing mental illness were included step-wise.

## Results

### Participants

A total of 1399 participants completed the survey. For a detailed analysis of the participants, see Table 1.

**Table 1:** Sample characteristics

Female gender	1206 (86.2)
Age	
Mean (SD)	40.1 (11.9)
Age range	18–85
Highest level of education (n, (%))	
No graduation	2 (0.1)
“Hauptschulabschluss” (year 9, lower secondary school certificate)	67 (4.8)
“Mittlere Reife” (year 10, lower secondary school certificate)/ Graduated from Polythechnical Highschool	319 (22.8)
A-Level Certificate	291 (20.8)
University degree	719 (51.4)
Other level of education	1 (0.1)
Living alone	210 (15.0)
Decrease of household income during CoVid-19	151 (10.8)
Systematically important work	757 (54.1)
Number of ACEs	
M (SD)	1.7 (1.9)
0 ACEs	480 (34.3)
1–3 ACEs	659 (47.1)
≥ 4 ACEs	239 (16.9)
Not stated	24 (1.7)
PHQ-8 before CoVid-19 (M, SD)	5.3 (3.5)
PHQ-8 during CoVid-19 (M, SD)	7.6 (5.0)
Pre-existing mental illness	477 (34.1)

*N* = 1399. Presented as n (%) or mean (M) (standard deviation (SD))

### Change of depressive symptoms

**Fig. 1:**
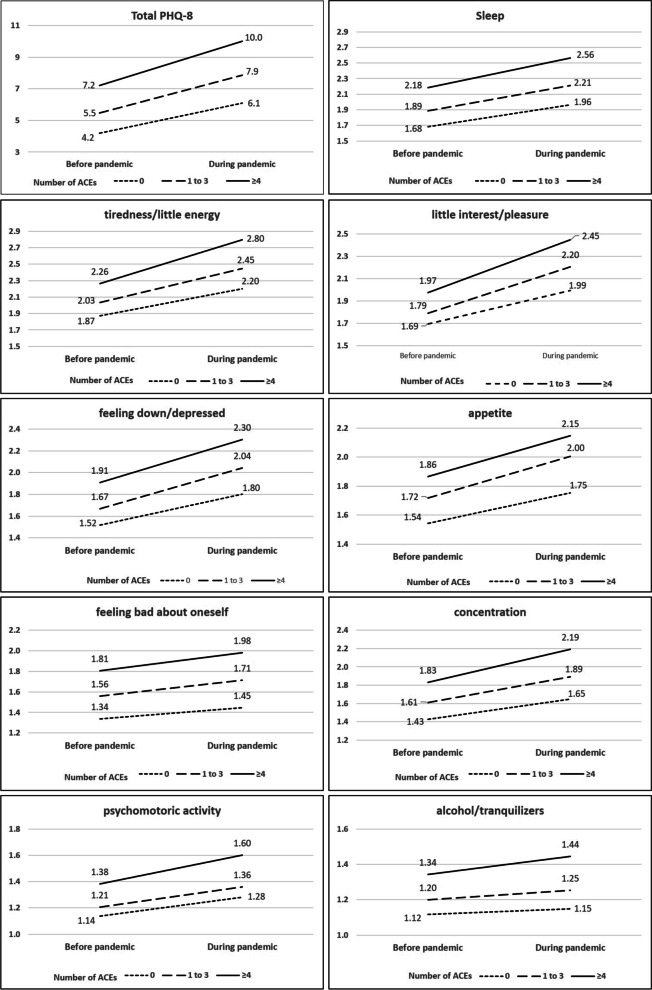
Capture: Depressive symptoms during Covid-19-pandemic. Repeated measure statistic for depressive symptoms during Covid-19-pandemic. A significant interaction effect between time and ACEs was seen for PHQ total score, tiredness/little energy, little interest/pleasure, trouble in concentration and resorting to alcohol/tranquilizers

The results of the two-way repeated measures ANOVA revealed that there was a significant main effect of time for all items. In detail, significant effects were seen for PHQ total score (*F* = 436.79, *p* < 0.001) and the items sleep (*F* = 211.87, *p* < 0.001), tiredness/little energy (*F* = 309.71, *p* < 0.001), little interest/pleasure (*F* = 289.25, *p* < 0.001), feeling down, depressed, hopeless (*F* = 159.06, *p* < 0.001), poor appetite or overeating (*F* = 175.06, *p* < 0.001), feeling bad about oneself (*F* = 83.86, *p* < 0.001), trouble in concentration (*F* = 229.75, *p* < 0.001) and moving/speaking slowly or fidgety/restlessness (*F* = 141.51, *p* < 0.001). Moreover, there was a significant main effect of time on resorting to alcohol/tranquilizers (*F* = 35.38, *p* < 0.001) (see Fig. 1).

### Severity of depressive symptoms in dependence of ACEs and income loss

Regarding ACEs, significant between-subject effects (in dependence of ACEs) were seen for PHQ total score (*F* = 71.26, *p* < 0.001) and the sub items sleep (*F* = 43.19, *p* < 0.001), tiredness/little energy (*F* = 45.65, *p* < 0.001), little interest/pleasure (*F* = 27.01, *p* < 0.001), feeling down, depressed, hopeless (*F* = 38.80, *p* < 0.001), poor appetite or overeating (*F* = 19.97, *p* < 0.001), feeling bad about oneself (*F* = 40.46, *p* < 0.001), trouble in concentration (*F* = 38.63, *p* < 0.001) and moving/speaking slowly or fidgety/restlessness (*F* = 23.42, *p* < 0.001). Moreover, there was a significant between-subject effect on resorting to alcohol/tranquilizers (*F* = 22.80, *p* < 0.001) (see Fig. 1).

Focusing on income loss, significant between-subject effects (in dependence of income loss) were seen for PHQ total score (*F* = 39.39, *p* < 0.001) and all assessed subitems: little interest/pleasure (*F* = 21.89, *p* < 0.001), feeling down, depressed, hopeless (*F* = 23.99, *p* < 0.001), poor sleep (*F* = 20.30, *p* < 0.001), tiredness/little energy (*F* = 14,85, *p* < 0.001), poor appetite or overeating (*F* = 10.22, *p* = 0.001), feeling bad about oneself (*F* = 28.55, *p* < 0.001), trouble in concentration (*F* = 15.92, *p* < 0.001) and moving/speaking slowly or fidgety/restlessness (*F* = 29.87, *p* < 0.001). Additionally, for resorting to alcohol/tranquilizers, there was a significant between-subject effect (*F* = 4.39, *p* = 0.036) (see supplementary material).

### Change of depressive symptoms in dependence of ACEs and income loss

For ACEs, a significant interaction effect between time and ACEs was seen for PHQ total score (*F* = 5.075, *p* = 0.006 with a mean of 4.2, 5.5 and 7.2 for 0, 1–3 and ≥ 4 ACEs before the pandemic and a mean of 6.1, 7.9 and 10.0 for 0, 1–3 and ≥ 4 ACEs during the pandemic). With regard to the items of the PHQ-8, significant interaction effects emerged for tiredness/little energy (*F* = 4.92, *p* = 0.007 with a mean of 1.78, 2.03 and 2.26, for 0, 1–3 and ≥ 4 ACEs before the pandemic and a mean of 2.20, 2.45 and 2.80 for 0, 1–3 and ≥ 4 ACEs during the pandemic), little interest/pleasure (*F* = 4.69, *p* = 0.009 with a mean of 1.69, 1.79 and 1.97, for 0, 1–3 and ≥ 4 ACEs before the pandemic and a mean of 1.99, 2.20 and 2.45 for 0, 1–3 and ≥ 4 ACEs during the pandemic), trouble in concentration (*F* = 4.04, *p* = 0.018 with a mean of 1.43, 1.61 and 1.83, for 0, 1–3 and ≥ 4 ACEs before the pandemic and a mean of 1.65, 1.89 and 2.19 for 0, 1–3 and ≥ 4 ACEs during the pandemic) and resorting to alcohol/tranquilizers (*F* = 3.15, *p* = 0.043 with a mean of 1.12, 1.20 and 1.34, for 0, 1–3 and ≥ 4 ACEs before the pandemic and a mean of 1.15, 1.25 and 1.44 for 0, 1–3 and ≥ 4 ACEs during the pandemic) (see Fig. 1).

Regarding income loss, a significant interaction effect between time and income loss was seen for PHQ total score (*F* = 27.84, *p* < 0.001 with a mean of 6.43 with and 5.20 without income loss before the pandemic and a mean of 10.28 with and 7.32 without income loss during the pandemic) and all assessed subitems: little interest/pleasure (*F* = 17.26, *p* < 0.001 with a mean of 1.89 with and 1.78 without income loss before the pandemic and a mean of 2.53 with and 2.13 without income loss during the pandemic) feeling down, depressed, hopeless (*F* = 20.68, *p* < 0.001 with a mean of 1.77 with and 1.64 without income loss before the pandemic and a mean of 2.38 with and 1.96 without income loss during the pandemic), poor sleep (*F* = 12.98, *p* < 0.001 with a mean of 2.03 with and 1.85 without income loss before the pandemic and a mean of 2.56 with and 2.14 without income loss during the pandemic), tiredness/little energy (*F* = 11.59, *p* < 0.001 with a mean of 2.11 with and 2.00 without income loss before the pandemic and a mean of 2.73 with and 2.39 without income loss during the pandemic), poor appetite or overeating (*F* = 5.67, *p* = 0.017 with a mean of 1.81 with and 1.67 without income loss before the pandemic and a mean of 2.19 with and 1.91 without income loss during the pandemic), feeling bad about oneself (*F* = 19.71, *p* < 0.001 with a mean of 1.73 with and 1.49 without income loss before the pandemic and a mean of 2.06 with and 1.62 without income loss during the pandemic), trouble in concentration (*F* = 15.96, *p* < 0.001 with a mean of 1.71 with and 1.58 without income loss before the pandemic and a mean of 2.18 with and 1.83 without income loss during the pandemic), and moving/speaking slowly or fidgety/restlessness (*F* = 10.98, *p* < 0.001 with a mean of 1.37 with and 1.19 without income loss before the pandemic and a mean of 1.66 with and 1.34 without income loss during the pandemic). For resorting to alcohol/tranquilizers, there was also a significant interaction effect between time and income loss (*F* = 9.99, *p* = 0.002 with a mean of 1.23 with and 1.19 without income loss before the pandemic and a mean of 1.37 with and 1.23 without income loss during the pandemic); (see supplementary material).

### Predictors for change of depressive symptoms

Results of the multiple linear regression indicated that there was a significant effect between age, sum of ACEs, systematically important work, decrease of household income, living alone, social support and pre-existing mental illness (*F* = 8.84, *p* < 0.001, R2 = 0.05) on change of depressive symptoms. At the individual level, a higher number of ACEs (t = -2.61, *p* = 0.009), and a decrease of household income (t = -4.08, *p* < 0.001) predicted an increase of depressive symptoms while age (t = 3.41, *p* = 0.001) and social support (*t* = 4.31, *p* < 0.001) were significant predictors for a decrease of depressive symptoms (see Table 2).

**Table 2:** Prediction of increase of change of depressive symptoms

	F (df)	*p-value*	R^2^	B	95% CI	*p-value*
**Model 1**	4.86 (2)	*0.008*	0.07			
Gender				0.26	(-0.33;0.84)	*0.393*
Age in years				0.03	(0.01;0.04)	*0.004*
**Model 2**	10.22 (3)	< *0.001*	0.02			
Gender				0.16	(-0.43;0.74)	*0.595*
Age in years				0.03	(0.01;0.05)	*0.001*
ACEs				-0.25	(-0.36;-0.14)	< *0.001*
**Model 3**	10.11 (7)	< *0.001*	0.05			
Gender				0.35	(-0.23;0.94)	*0.237*
Age in years				0.03	(0.01;0.05)	*0.001*
ACEs				-0.16	(-0.27;-0.04)	*0.009*
Systematically important work				0.01	(-0.28;0.30)	*0.942*
Decrease of household income				-1.36	(-2.01;-0.70)	< *0.001*
Living alone				-0.22	(-0.79;0.35)	*0.453*
Social support				0.21	(0.11;0.30)	< *0.001*
Pre-existing mental illness				-0.15	(-0.60;0.30)	*0.519*

Regression analysis, presented as unstandardized coefficient B and 95% CI = 95% Confidence Interval

## Discussion

To the best of our knowledge, this is the first study assessing the role of ACEs on the change of depressive symptoms during the Covid-19 pandemic. Importantly, the experience of adversity during childhood and/or adolescence is associated with an increase of depressive symptoms. Therefore, our results identify persons who have experienced adversity during childhood or adolescence as a risk group for mental health problems during the pandemic. This has been hypothesized before [19], as personss with ACEs have a higher sensitivity to stress and threat and lower coping abilities, resulting in a higher vulnerability to stress exposure [32, 33]. While it was shown before that ACEs are associated with higher depressive symptoms during the pandemic [10, 34] and higher post-traumatic stress symptoms if they or someone in the family, neighborhood or among friends have suffered from Covid-19 [41], this study is the first to confirm a particular vulnerability for subjects with ACEs for an increase of depressive symptoms during the current pandemic in Germany.

Besides the relevance of ACEs, our findings show an increase of depressive symptoms in all participants. This finding is in line with studies indicating an increase of depressive symptoms during the pandemic [8, 16, 42, 43]. Our findings thereby substantiate results on the mental health burden during the current pandemic.

Additionally, the data presented show that ACEs where associated with higher depressive symptoms in a dose dependent manner. This association was shown already more than 20 years ago in the famous ACE study [27] and has been confirmed also in German population representative samples [44].

In a next step, we assessed further predictors for an increase of depressive symptoms during the pandemic, were ACEs were shown to be associated with a stronger increase in depressive symptoms. In line with this finding, ACEs were shown to be associated with a stronger decrease in quality of life and general health status during the pandemic [37, 45]. Moreover, ACEs we have demonstrated that ACEs are associated with dysfunctional familiar coping strategies during the pandemic [35, 36] and a higher risk for intrafamiliar problems [35, 37, 38]. The reason for this seen higher risk of problems during the pandemic may be manifold. A higher stress-vulnerability and a decrease of emotion regulation [32, 33] was shown for individuals having experienced ACEs, potentially affecting coping of stressful situations. ACEs affect stress regulation via one main stress axis of the body, the hypothalamic–pituitary–adrenal (HPA) axis in the long-term [46]. Consequently, cortisol metabolism is known to be altered in adults who have experienced ACEs [47]. This may contribute to the higher vulnerability of persons who have experienced ACEs during the stressful times of a pandemic. In a population-based sample of the German population, a significant impact of ACEs and stressful life events during adulthood, such as, death of a loved one, was demonstrated. Nevertheless, no significant interaction was shown, meaning that ACEs did not modify the association between major stressful life events in adulthood and health [48]. However, the interplay between ACEs and stressful life events in adulthood is not well explored and should be topic of further research.

Beside ACEs, lack of social support was associated with a significant increase of depressive symptoms. Loneliness has been identified before as major mental health concern during the pandemic [12]. Living alone, on the other hand, did not predict an increase in depressive symptoms, indicating that social support is more relevant for an increase of depressive symptoms, whether someone lives alone or not.

While a systemically important work was not a significant predictor, our results show that a decrease of income is associated with a higher risk for increased depressive symptoms. The role of economic hardship on mental health has been shown numerously [49–51]. In a recent cross-sectional online survey in an Austrian sample, no work and low income were predictors for higher symptoms of depression and anxiety during the pandemic [16], just as discontinued working activity in an Italian sample [43] and economic stressors in a US sample [15]. ACEs are known to increase the risk for a lower household income, but also financial wellbeing and financial literacy [31]. The modifying effect of income loss on change of depressive symptoms during the pandemic was even higher compared to ACEs. This points towards the relevance of financial security for mental health during crisis such as the pandemic. However, the association between income loss and increased risk for depressive symptoms as well as an increase of depressive symptoms may be more complex. Besides the financial aspect, other factors encompassing job loss and thus loss of structure, social contacts and appreciation may play a role in the observed increase of depressive symptoms. However, as in our study besides income loss no further assessments were conducted, further studies are necessary in order to disentangle this complex field.

Gender had no significant impact on increase of depressive symptoms during the pandemic. Although depression is more frequent in females, studies assessing depression during the pandemic reveal contradicting results for the role of gender [9, 16]. However, as the number of male participants in our sample has been very low, this may impair the validity of this result. Our data reveal younger age as a significant predictor of an increase of depressive symptoms during the pandemic, which is in line with the literature [10, 52].

One major limitation of the study is that the participants cannot be considered as representative for the general public as a non-probability sample based on participation in an online survey was used. Compared to the general population in Germany, our sample comprised far more females, was younger [53], academic achievement was higher [54] and less subjects in our sample lived alone [55]. This bias may have occurred due to our ways of recruitment (our homepage, social media and print media and existing mailing lists from other studies and interested parties). These methods may have prioritized the academic, and in particular the medical field, as our homepage is a clinical one and the majority of persons on our mailing list are somehow related to the medical field As the impact of economic loss was already highly significant in our well educated sample, it can be assumed that the impact of income loss may be even more relevant in a sample with a more representative socioeconomic status. A similar bias may be assumed for ACEs. Lower education correlates with higher rates of ACEs [56, 57]. Consequently, the results in our sample may underestimate the negative impact of ACEs during the pandemic. Together, the generalizability of our findings is limited. The assessment of depressive symptoms before the pandemic are based on retrospective self-report. Importantly, recall bias was shown to diverge between depressed patients and healthy controls in a complex way [58], why the validity of the retrospectively assessed depressive symptoms prior to the pandemic is reduced. The use of retrospectively assessed ACEs is debated [59]. However, the relevance of subjective adversity for health was underlined recently [60]. In our cross-sectional study, causality cannot be deduced. Due to reasons of feasibility, some items were merged, e.g. the use of alcohol and tranquilizers. However, the presented results give a meaningful first insight into the relevance of ACEs on coping with the pandemic.

## Conclusion

ACEs are a significant predictor for an increase in depressive symptoms during the pandemic, indicating that subjects with ACEs may be a risk group for mental health problems during the current and potential later pandemics. An even stronger association was found between income loss and increase of depressive symptoms. Other predictors are young age and lack of social support.

These findings underline the relevance of interventions against social isolation, economic loss and the need of mental health services during pandemics. To know about the increased risk for people with ACEs during the pandemic may help to provide more targeted support in possible scenarios due to the current or possible other pandemics. Economic stability seems to be of prior importance for mental health.

## Supplementary Information

**Additional file 1.**

## Data Availability

The datasets used and analyzed during the current study are available from the corresponding author on reasonable request.
