# Supplementary material for: Adverse childhood experiences are associated with a higher risk for increased depressive symptoms during Covid-19 pandemic – a cross-sectional study in Germany
Source: BMC Psychiatry. 2022 Aug 11;22:540. doi: 10.1186/s12888-022-04177-7 (PMC9365680; doi:10.1186/s12888-022-04177-7)
Supplement: Supplementary file 1 — Additional file 1. [file 12888_2022_4177_MOESM1_ESM.docx]

**Annex**


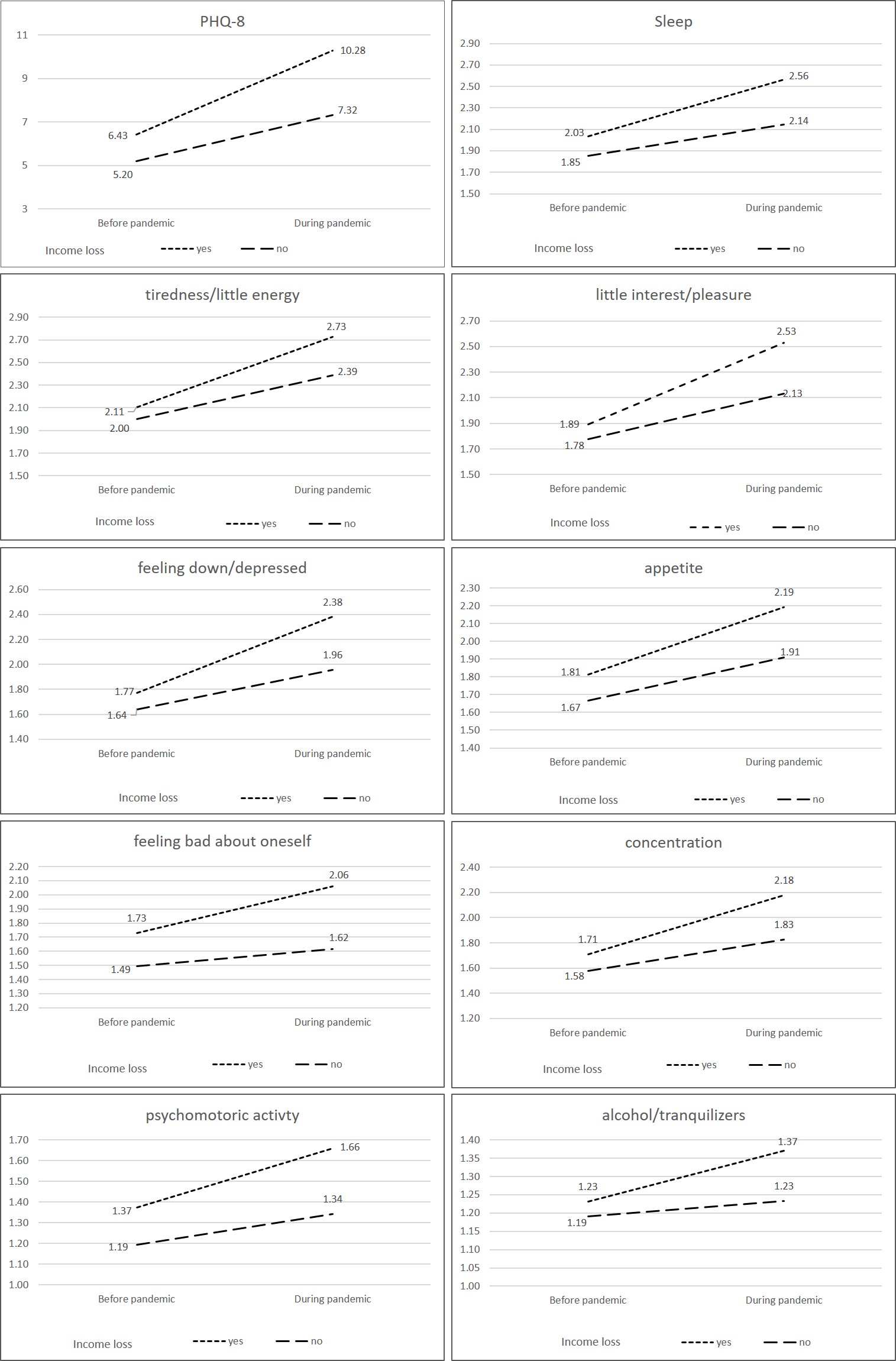


Repeated measure statistic for depressive symptoms during Covid-19-pandemic. A significant interaction effect between time and income was seen for PHQ total score, as well as all assessed subitems and resorting to alcohol/tranquilizers.
